# Ring cell migration assay identifies distinct effects of extracellular matrix proteins on cancer cell migration

**DOI:** 10.1186/1756-0500-7-183

**Published:** 2014-03-27

**Authors:** Hui Chen, Josephine Nalbantoglu

**Affiliations:** 1 Division of Experimental Medicine, Department of Medicine, McGill University, Montreal Quebec, Canada; 2 Department of Neurology & Neurosurgery, McGill University, Montreal Quebec, Canada; 3 Montreal Neurological Institute, McGill University, 3801 University Street, Montreal, Quebec H3A 2B4, Canada

**Keywords:** Cell migration assay, Cloning ring, Extracellular matrices

## Abstract

**Background:**

Alterations in cell migration are a hallmark of cancer cell invasion and metastasis. *In vitro* assays commonly used to study cell migration, including the scratch wound healing assay, Boyden chamber assay, and newly developed advanced systems with microfluidics, each have several disadvantages.

**Findings:**

Here we describe an easy and cost-effective *in vitro* assay for cell migration employing cloning rings to create gaps in the cell monolayer (“ring cell migration assay”). The assay was used to quantitate innate differences in cell motility and the effect of various extracellular matrix proteins on migration of five cancer cell lines: U87 and U251N glioma cells, MDA-MB-231and MCF-7 breast cancer cells, and HeLa cervical cancer cells. Interestingly, collagen was a general promoter of cell migration for all five cancer cell lines, without affecting cell proliferation.

**Conclusions:**

Taken together, the ring cell migration assay is an easy, convenient and cost-effective assay to study cell migration *in vitro*.

## Findings

### Introduction

Metastasis is the leading cause of mortality for patients suffering from cancer. During metastasis, abnormal cells with genomic heterogeneity may undergo epithelial-to-mesenchymal transition (EMT) and phenotypically exhibit increased motility and invasiveness as compared to normal cells [1-3]. Thus, increased cell migration is a priming process for cancer cells to invade and metastasize during cancer progression. Therefore, evaluating cancer cell motility is a critical step in studying mechanism of cancer cell invasion and metastasis.

Extracellular matrix (ECM) proteins provide a structural basis supporting cell migration; they can also either function as a reservoir for growth factors, or directly bind to receptors such as integrins to activate cellular signaling pathways specifically driving cell migration [4-6]. Therefore, ECM plays critical roles in cancer cell invasion and metastasis [7-10]. However, it is mostly unknown how various ECM proteins affect cell migration of different tissue-specific cancer cell types.

The different techniques used to assess cell migration and invasion have been described and critically evaluated in recent review articles [11,12]. There are several conventional *in vitro* assays for studying cancer cell migration, including scratch wound healing assay [13] and Boyden chamber assay [14]. While these assays have advantages either in ease of performance (scratch assay) or in mimicking *in vivo* chemoattractant gradients for cell migration (Boyden assay), they also have many disadvantages. For example, scratch wound healing assay is not applicable to every type of cancer cell as some monolayers remain hard for scratching and cells may be damaged during the wounding, while Boyden chamber assay is difficult to reproduce as it is dependent on the number of cells seeded and only provides endpoint data of cell migration. Additional assays have been configured to overcome some of these problems, such as the cell exclusion zone assay in which cells are cultured on microfabricated stencils [15] or in the presence of silicone stoppers which are removed at cell confluence [16], generating cell-free areas with well-defined linear borders. However, microfabrication is not available to all laboratories, and care must be taken to prevent cell entry under the stopper when silicone inserts are used. Thus, an easy, convenient and cost-effective assay with a high level of reproducibility is required for rapid assessment of cell migration.

Here we describe an easy assay for cell migration, based on the principles of the cell exclusion assay, and which we have termed “ring cell migration assay” as it uses a cloning ring to establish the gap between two parts of a monolayer. We provide detailed procedures to perform the assay. We tested five cancer cell lines of different tissue origin to verify the assay’s ability to distinguish differences in cancer cell motility and to analyze the effect of various extracellular matrix proteins on cancer cell migration.

### Materials and methods

#### Cell culture and reagents

Two glioma cell lines U87 and U251N, two breast cancer cell lines MDA-MB-231 and MCF-7, and HeLa cervical cancer cells were obtained from American Type Cell Collection (ATCC) (Manassas, VA) and routinely maintained in Dulbecco’s modified Eagle’s medium (DMEM) with 10% fetal bovine serum (FBS) supplemented with 100 μg/ml of penicillin-streptomycin. Pyrex cloning rings of 8 mm × 8 mm (Catalog No. CLS 31668-125EA) used in the ring cell migration assay were made by Corning and were purchased from Sigma-Aldrich. The XTT (2,3-Bis-(2-Methoxy-4-Nitro-5-Sulfophenyl)-2H-Tetrazolium-5-Carboxanilide) (Catalog No. X4251) used in cell proliferation assay was purchased from Sigma-Aldrich. The sources and coating concentrations of ECM proteins are listed in Table 1.

**Table 1:** ECM proteins used in this study

**ECM**	**Stock concentration**	**Final concentration**	**Source**
Poly-D-lysine	1 mg/ml	50 μg/ml	Sigma P7280
Type I collagen	2.5 mg/ml	100 μg/ml	Sigma C9791
Fibronectin	1 mg/ml	20 μg/ml	Sigma F1141
Vitronectin	50 μg/ml	2 μg/ml	Sigma V8379
Laminin	0.9 mg/ml	10 μg/ml	Invitrogen 23017-015

ECM proteins were added at the indicated final concentration in a volume of 500 μl, and left overnight at 4°C.

#### Ring cell migration assay

Before use, the rings preserved in alcohol solution were briefly flamed using tweezers. The rings were placed vertically in each well of six-well plates, either coated with ECM proteins or not, with a maximum of three rings in each well keeping appropriate distance from each other. Cell suspensions were added carefully to both sides of each ring. Depending on the cell line, the cell number varied between 5,000 and 10,000 cells inside the ring, and between 250,000 to 300,000 cells outside the ring, reaching about 80% confluency when cells were attached. After cells attached firmly, which may take four to eight hours, the rings were carefully removed straight up using tweezers followed by washing with 1 × Phosphate-Buffered Saline (PBS) and preserving in alcohol solutions for future uses. The cells were washed gently with PBS to remove cell debris while avoiding detachment of the cells. The cells were replenished with fresh DMEM containing 10% FBS and 100 μg/ml of penicillin-streptomycin. The gaps of the cell rings were observed under microscope (Leica Microsystems, Wetzlar, Germany) and photographs were taken at various, marked sites along the rings at the start of assay (T0); between 4 and 6 positions were marked for imaging per ring. At different time points, e.g. 24 (T1) or 48 (T2) hours, photographs were taken again of the remaining gap, at the same pre-specified positions along the rings as at T0.

#### Image analysis

The areas of the gaps were measured using Image J software (National Institutes of Health, Bethesda, MD). The pictures of gaps taken at different time points were opened in Image J. The distance pixels were set as “400” and the unit of length was set as “mm”. The shapes of the gap areas were selected using the “freehand selections” and the area was measured.

#### XTT proliferation assay

Five thousand cells were plated into each well of 96-well plates coated with or without indicated ECM protein. The cells were kept in the 37°C incubator supplied with 5% CO_2_. After 24, 48 or 72 hours incubation, the medium of cells was removed and the cells were washed with 1 × PBS. Fifty microliters of XTT solution were added to each well and the plates were kept in the 37°C incubator for four hours. The O.D. (450 nm) of each well was determined by Multiskan EX Microplate Photometer (Thermo Scientific, Waltham, MA).

#### Statistical analysis

The differences in cell migration due to the effects of the different ECM proteins were determined by one-way ANOVA analysis using Prism software (GraphPad). The difference was considered as significant when p < 0.05 after Bonferroni correction for multiple testing.

### Results

#### Set-up for the ring cell migration assay

**Figure 1:**
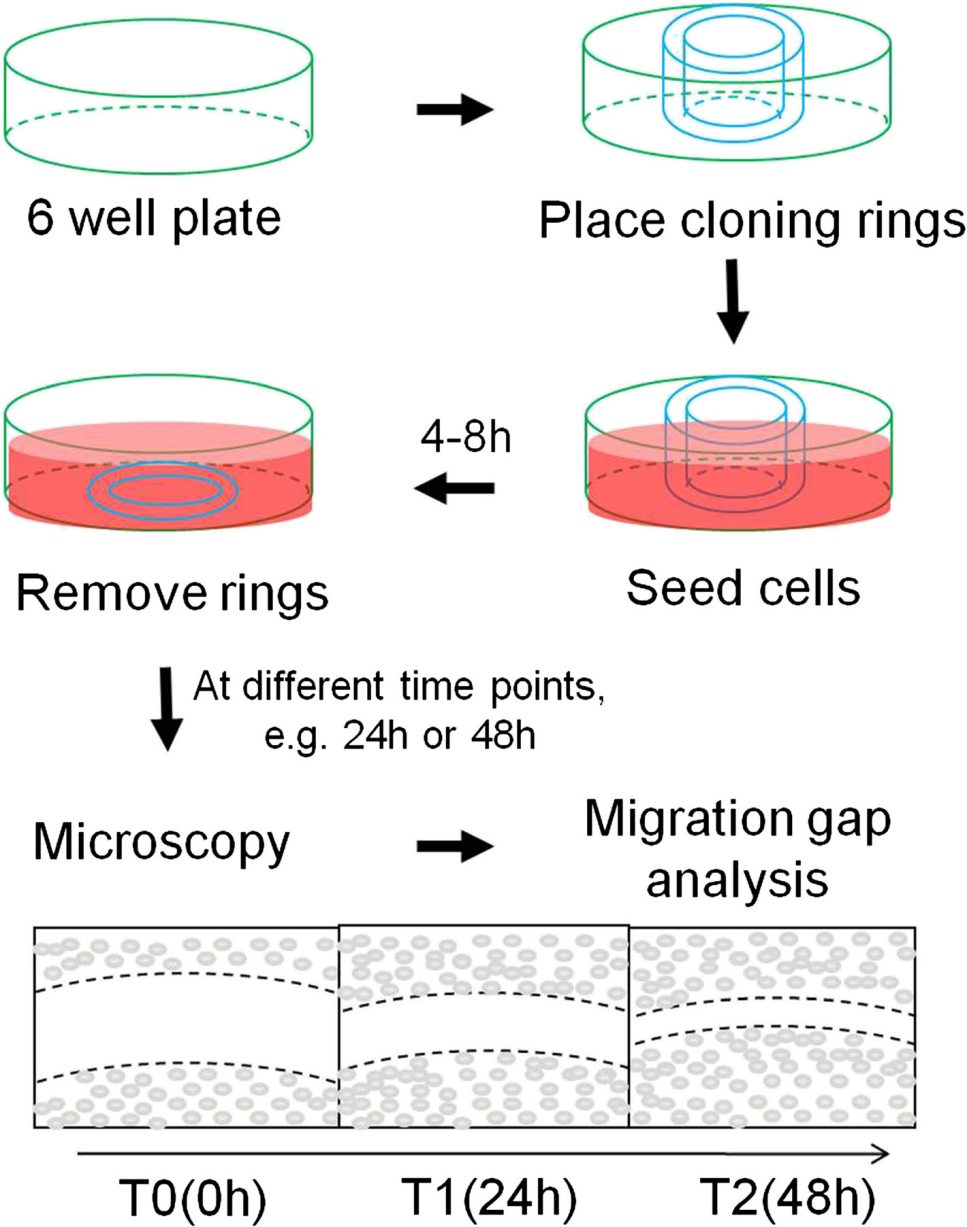
**Schematic representation of ring cell migration assay.** Cloning rings were used as stoppers after cells were seeded. Removal of rings created ring gaps which allow cells to migrate. The migration of cells was monitored at various time points and the closure rate of the gap was measured to compare the motility of cells.

**Figure 2:**
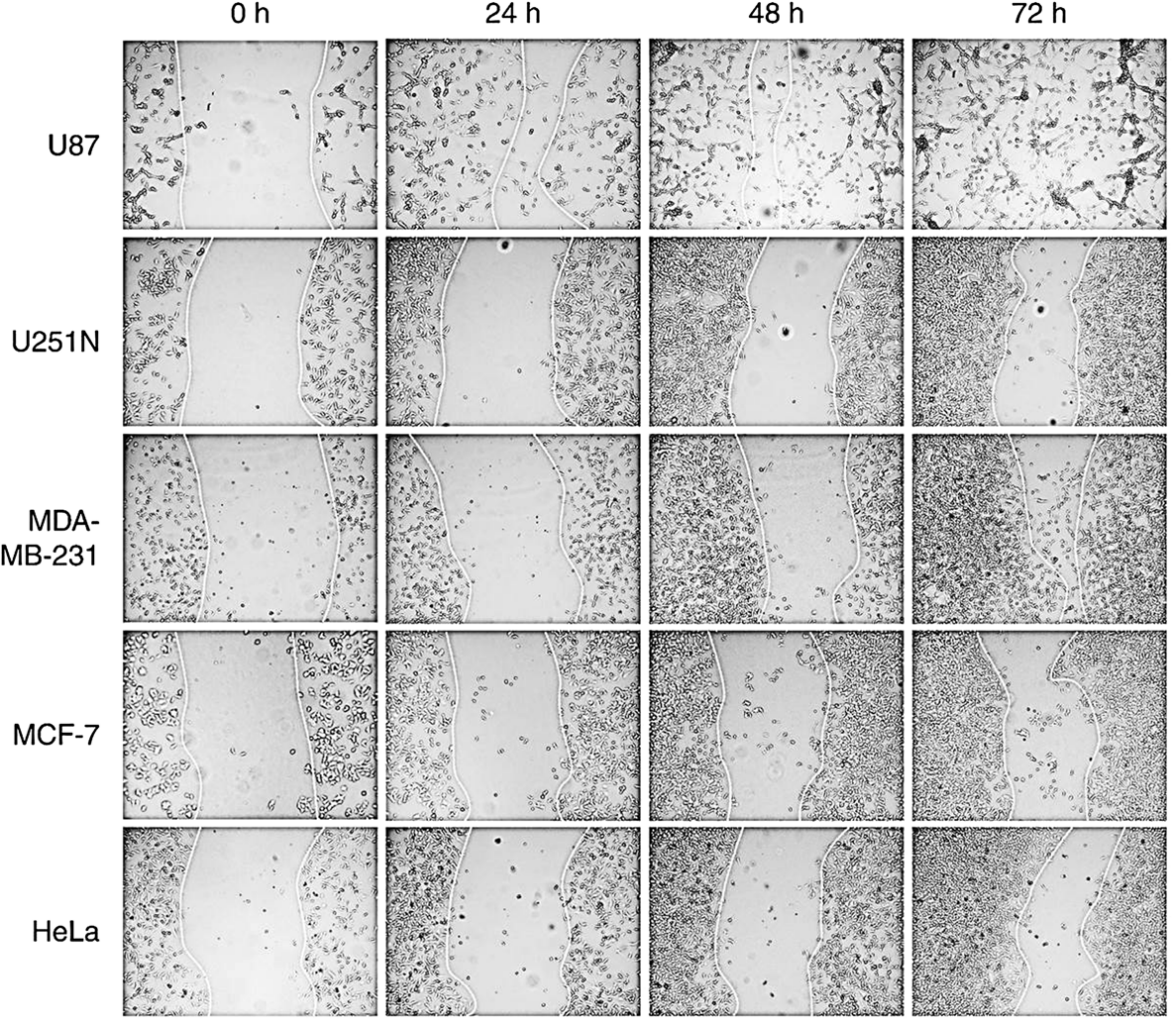
**Cancer cell migration using ring cell migration assay.** Cells (250,000 to 300,000) were seeded in 6-well plates with cloning rings. After removal of rings, photographs were taken of the gaps observed under microscope at different time points, i.e. 0 h, 24 h, 48 h and 72 h. The dotted lines delineate the migrating edges of cells.

Although simple and easy, the conventional scratch assay has been shown to be very difficult in generating good and reliable scratches for many cell lines, including U87 cells. To keep the advantage and to overcome the shortcoming, we sought to develop an easy assay for cell migration. As described in Materials and Methods and shown in Figure 1, the ring cell migration assay constitutes a very simple set-up of experiments by using cloning rings, which are placed before seeding cells and removed after cells attached. The gaps formed before cells migrated allowed all cell lines to be analyzed using this assay. Although the cloning rings create ring-shaped gaps, it was difficult and inconvenient to monitor the entire gap under the microscope. Instead, we marked part of the ring at various sites and tracked them at various time points, mimicking an exclusion zone assay (Additional file 1: Figure S1). This allowed us to obtain more data points from single rings when each part of the ring gap was measured. In addition, we found that data points in each experiment could be increased by placing up to three rings in each well of six-well plates, making the assay more reproducible and efficient. We found that cell confluency did not significantly affect the migration rate of cells (data not shown) and that the difference in cell motility of the different cell lines was independent of cell confluency; for example, U87 cells at lower confluency migrated faster than U251N cells did at higher confluency (Figure 2), suggesting that cell motility is an intrinsic property of migrating cells. Thus, the ring cell migration assay is a simple, cost-effective and efficient way to analyze motility of cells.

#### Cancer cell motility as visualized during ring cell migration assay

To test whether ring cell migration can distinguish differences in cell motility among different cell lines, two sets of cancer cells, i.e. glioma cells (U87 and U251N) and breast cancer cells (MDA-MB-231 and MCF-7), with different migratory potential were used in this assay and compared to HeLa cells. All five cell lines were processed in the ring cell migration assay as described above. At different time points, i.e. 0 h, 24 h, 48 h and 72 h, photographs were taken of the remaining gap from the same sites along the ring. As shown in Figure 2, while the gap area of all five cell lines was the same at the start of the assay (indicating the uniform thickness of the cloning ring), the rate of closing of the gaps differed significantly with increasing time. It was clear that the U87 glioma cells migrated faster than U251N cells even at 24 h, and more so at the 72 h time point. Similarly, the MDA-MB-231 breast cancer cells migrated faster than MCF-7 cells. Thus, the ring cell migration assay can be used to distinguish the motility of different cancer cell lines.

#### Quantitation of cancer cell migration using the ring cell migration assay

**Figure 3:**
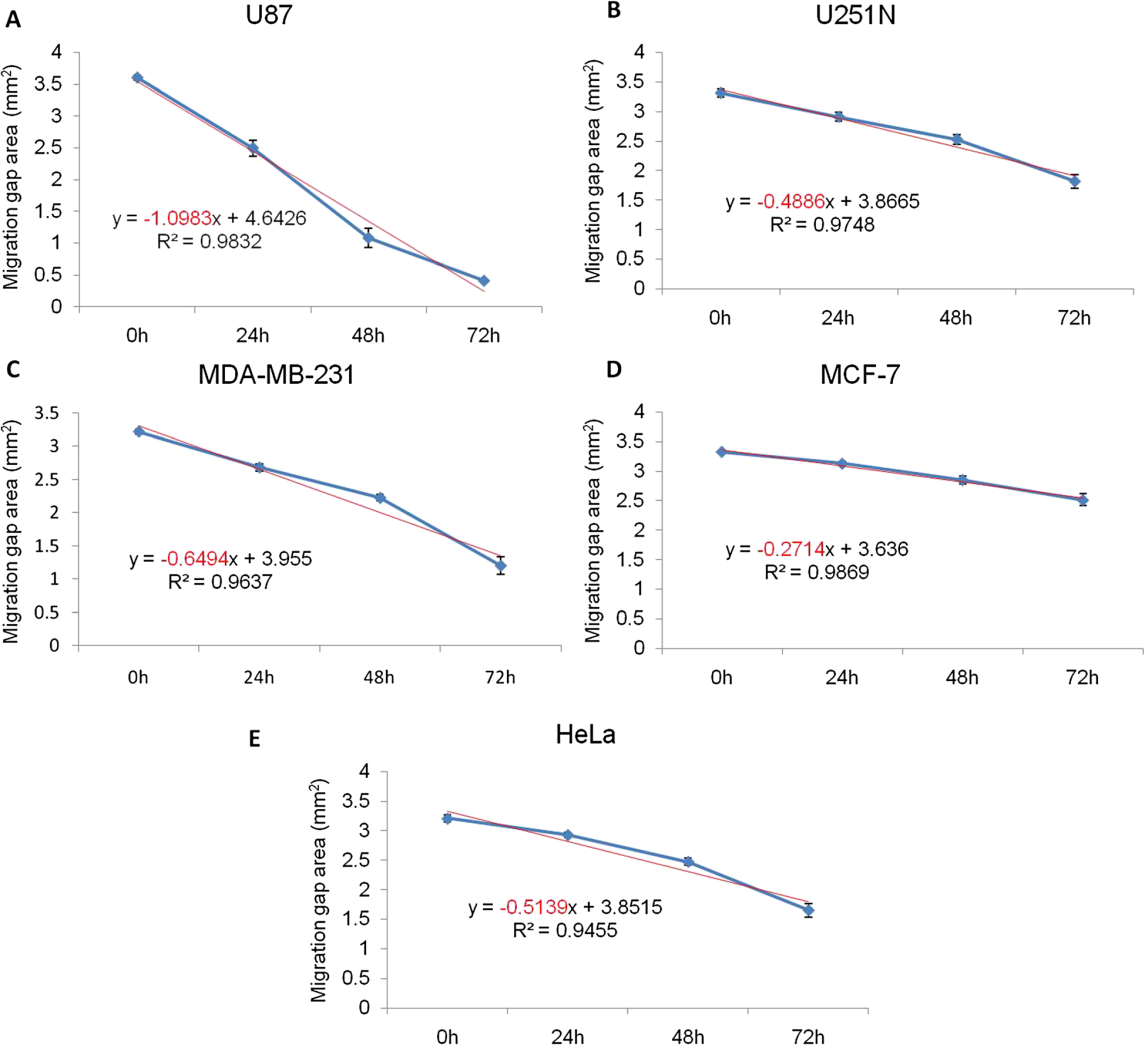
**Motility of different cancer cells in ring cell migration assay. (A)** U87 cells, **(B)** U251N cells, **(C)** MDA-MB-231, **(D)** MCF-7, **(E)** HeLa cells. The gap areas photographed during ring cell migration assays from Figure 2 were measured using Image J software. The areas were plotted against migration time. For each plot, an equation and a R^2^ value were given. The slope (mm^2^/h) was used to indicate the difference in cancer cell motility. Values represent the mean ± s.e.m. (some of which are obscured by the symbols). N = 5 to 11 gaps from three rings. Data shown are representative of three independent experiments.

Data obtained from photomicrographs such as those shown in Figure 2 can yield quantitative comparisons between cell lines. To do so, a group of gap areas from the same sites at individual time points were measured and plotted against time, and a trend line and a R^2^ value were calculated for each plot. As shown in Figure 3, the slope of the trend line for U87, U251N, MDA-MB-231, MCF-7 and HeLa cells was −1.0983, −0.4886, −0.6494, −0.2714 and −0.5139 mm^2^/h respectively. Thus, the motility of each cell line could be compared according to the slope value, i.e. U87 > MDA-MB-231 > HeLa > U251N > MCF-7 cells. Therefore, the motility of cancer cells can be quantified and compared in the ring cell migration assay.

#### Effect of ECM proteins on cancer cell migration

**Figure 4:**
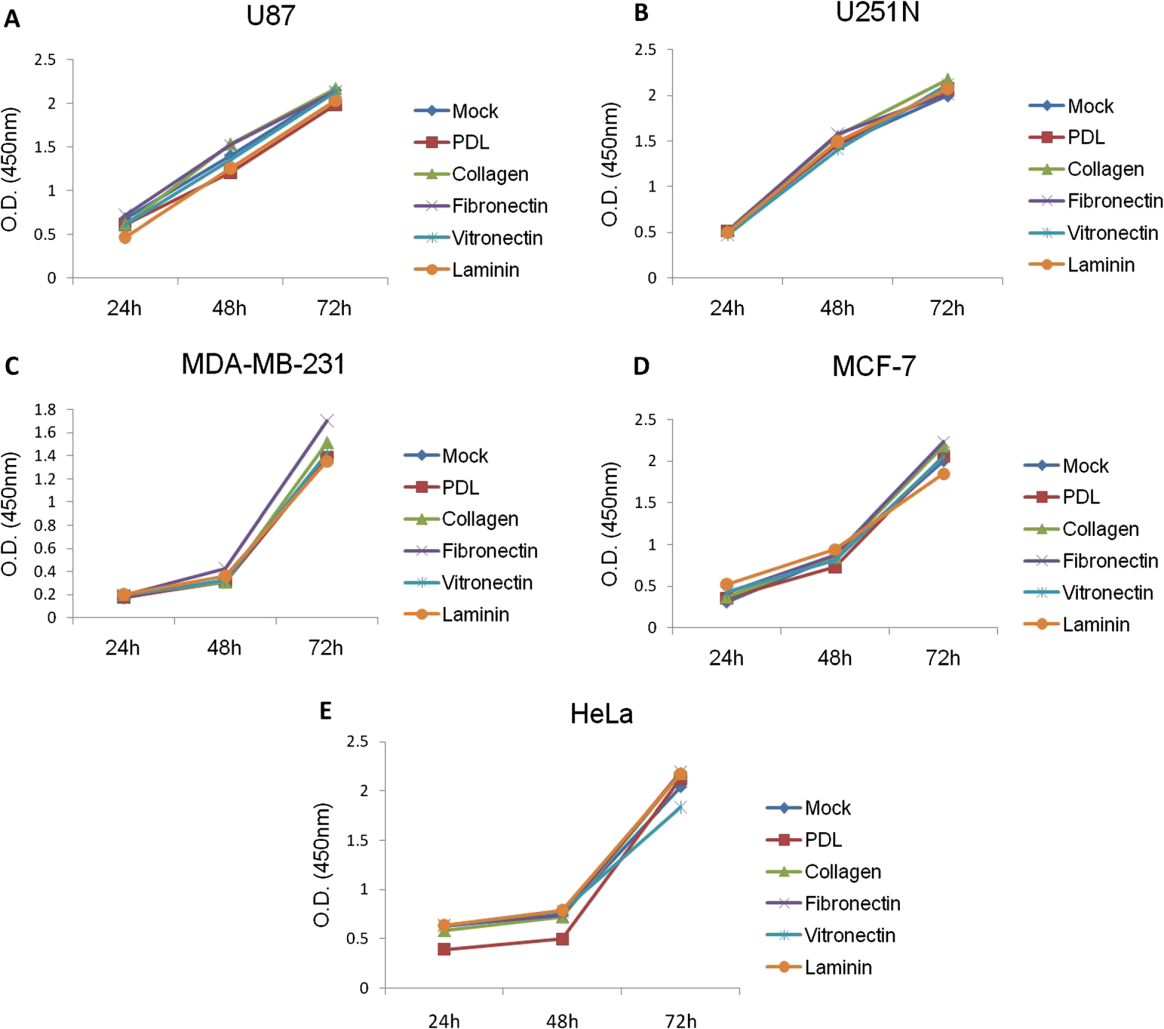
**Effect of ECM proteins on cancer cell proliferation. (A)** U87 cells, **(B)** U251N cells, **(C)** MDA-MB-231, **(D)** MCF-7, **(E)** HeLa cells. Cells (5,000) were seeded in 96 well-plates coated with various ECM proteins. XTT solution was added to cells at different times in culture, i.e. 24 h, 48 h and 72 h. The metabolic activity of the cells was measured at 450 nm using Ascent software. Values represent the mean for three replicates. Data shown are representative of three independent experiments.

Next, we assessed whether this newly developed cell migration assay could also be applied to evaluate how ECM proteins affect 2-dimensional migration. To exclude the possibility that ECM proteins may affect cell proliferation in the short term, thereby influencing cell migration results, the different cell lines were cultured on the various ECM proteins and their proliferation rate was analyzed. As shown in Figure 4, all five cell lines used in this assay showed continuous growth up to 72 h, and no significant difference was found in presence of the ECM proteins at individual time points, suggesting that these did not affect the proliferation of the cancer cells. Since there were no differences when proliferation assays were carried out with cultures that were subconfluent at the beginning, it can be presumed that differences in proliferation rate will not influence the results of the ring migration assay in which the cell monolayer is more likely to be confluent and inhibited by contact.

**Figure 5:**
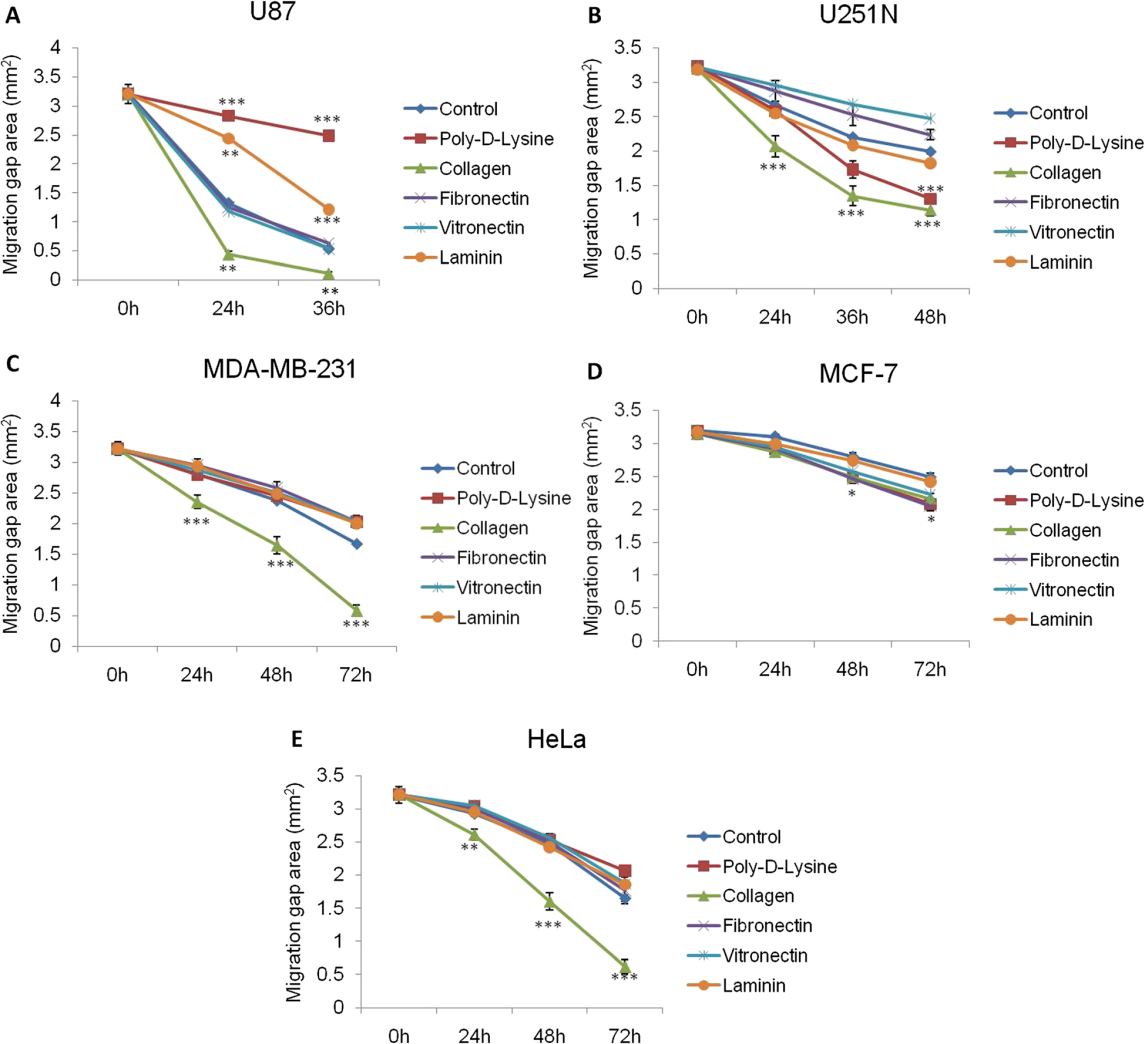
**Effect of ECM proteins on cancer cell migration. (A)** U87 cells, **(B)** U251N cells, **(C)** MDA-MB-231, **(D)** MCF-7, **(E)** HeLa cells. Cells (250,000 to 300,000) were seeded in 6-well plates pre-coated with various ECM proteins and cloning rings were used as described in Materials and Methods. After removal of rings, photographs of the gaps were taken at different migration time points, i.e. 0 h, 24 h, (or 36 h), 48 h and 72 h. The gap areas of the ring cell migration assays were measured using Image J software. The areas were plotted against migration time. Values represent the mean ± s.e.m. (some of which are obscured by the symbols). N = 4 to 10 gaps from three rings. Data shown are representative of three independent experiments. The differences at each time point between control and various ECM protein coatings were determined by post-hoc tests for pairwise comparison (after one-way ANOVA analysis) and Bonferroni correction for multiple testing. *p < 0.05; **p < 0.01; ***p < 0.001.

To evaluate the effect of ECM proteins on cancer cell migration, various proteins were each coated onto the plates before performing the ring cell migration assays. As shown in Figure 5 and Additional file 2: Figure S2, while each cancer cell line exhibited a different pattern of cell migration on various ECM proteins, collagen appeared to be a general promoter of migration for all these cell lines. Although laminin had no effect on the migration of other cell lines, it significantly inhibited U87 cell migration. Interestingly, while poly-D-lysine inhibited U87 cell migration, it promoted U251N cell migration significantly at 72 h. These findings illustrate the utility of this assay on assessing the effect of ECM components on short-term cell migration.

### Discussion

We present a newly developed, simple and quantitative *in vitro* cell migration assay that can also be applied to evaluate the effect of various extracellular matrix components on coated plates. This assay is a variation of the cell exclusion zone assay whereby a cell-free zone is created without disturbance of the cell monolayer. The most critical steps in this assay are the placement and removal of the cloning rings before and after cell seeding, respectively. As shown in Figure 2, differences in cell motility of the various cancer cell lines can be observed at 24 h or even earlier (data not shown), suggesting that this assay may be adapted to shorter time periods, especially when combined with time-lapse videomicroscopy.

Compared to other conventional and advanced cell migration assays, this assay has many advantages. Removal of the cloning ring after cells have attached to the inside and outside compartments produces a uniform gap and does not damage the cells, or the underlying extracellular matrix, in contrast to the scratch wound healing assay [13] which may lead to cell death and release of cellular factors during the scratch process. As well, the ring migration assay can be applied to cells which do not produce ideal scratches (such as the U87 glioma cells) and scratch-refractory monolayers. Other individually-developed assays for analyzing cell migration include the circle wound-healing assay [17] which uses a drill bit to make round-shaped wounds in a cell monolayer, and suffers from many of the same limitations as the scratch wound healing assay. Even the more advanced techniques such as the electric cell-substrate impedance sensing method [18] which produces uniform gaps in the cell monolayer with a pulse of high current still lead to cell death, producing cell remnants that may interfere with proper wound closing.

While the Boyden chamber assay [14] provides a chemotactic gradient as cells migrate through the pores of a transwell, only one data point can be obtained at the very end of the experiment, and cell migration cannot be visualized in real time, in contrast to the ring migration assay in which cell movement can be observed by time-lapse videomicroscopy (data not shown). Furthermore Boyden assays are more costly because commercially available transwell plates must be used instead of the much cheaper cloning rings which can be recycled many times. New developments include microfluidics-based cell migration apparatuses that use microchannels with mixed flow of microfluids to study cell movement [19]. In the microfluidic system, cells are seeded in microchambers and streams of media with and without chemoattractants are applied in parallel. Combined with time-lapse microscopy, these assays provide insight into the migration behavior of cells, allowing single cells to be tracked and the direction and velocity of each cell to be calculated. However, these sophisticated systems are expensive and may be unaffordable for many labs.

We took advantage of the ability to obtain quantitative measures to study the effect of ECM proteins on the migration of the different cell lines. The ECM constitutes an important component of the microenvironment during cancer cell invasion and metastasis [8,9]. Collagen is known to play a critical role in tumorigenesis [20] and angiogenesis [21].Our results suggest that collagen functions as a general promoter for glioma, breast and cervical cancer cell migration (Figure 5). In contrast, Ohtaka et al. showed that laminin had a stronger impact on promoting colon cancer cell migration than other extracellular matrix proteins such as collagen [22], while pancreatic cancer cell migration was positively influenced by collagen, as well as fibronectin and laminin [23]. These studies point to the diverse roles that ECM proteins play in regulating migration of tissue-specific cancer cells. Interestingly, collagen, as well as other ECM proteins, did not affect the proliferation rate of any of the cancer cell lines used here (Figure 4) but had distinct effects on cell migration (Figure 5), suggesting that these proteins may activate different signaling pathways specific for cell migration, such as signaling though integrins [6]. The observed differences on the effect of ECM proteins on the migration of the various cell lines might be due to tissue-specific expression of receptors that bind to and are activated by different ECM proteins [4].

In summary, the ring cell migration assay is an easy, cost-effective method for quantitative analysis of cell migration *in vitro*. It is adaptable to both short and long-term evaluation periods using time-lapse microscopy. Although the assay as described here does not involve chemotactic gradients, and is limited to analyzing autonomous cell migration, modifications can be envisaged, for example, through implantation of matrigel containing chemokines within the ring, in order to create the chemotactic gradient. Thus, the ring migration assay may have broad applications in different fields.

## Abbreviations

ANOVA: Analysis of variance; ATCC: American Type Cell Collection; DMEM: Dulbecco’s modified Eagle’s medium; EMT: Epithelial-to-mesenchymal transition; ECM: Extracellular matrix; FBS: Fetal bovine serum; O.D.: Optical density; PBS: Phosphate-buffered saline; XTT: 2,3-Bis-(2-Methoxy-4-Nitro-5-Sulfophenyl)-2H-Tetrazolium-5-Carboxanilide.

## Competing interests

The authors declare that they have no competing interests.

## Authors’ contributions

HC and JN conceived and designed this study. HC carried out experiments and collected data. HC and JN analyzed data and wrote the manuscript. Both authors read and approved the final manuscript.

## Supplementary Material

Additional file 1: Figure S1 Representation of the gap of the U251N glioma cells at 24 h after removal of the cloning ring. Click here for file

Additional file 2: Figure S2 Slope as an indicator of distinct effects of extracellular matrices on cell migration. Ring cell migration assay was performed on U87, U251N, MDAMB231, MCF7 and HeLa cells grown on the indicated extracellular matrices and the slope was measured from Figure 5. One-way ANOVA Dunnett’s Multiple Comparison Test: Compare all columns vs. Control, N = 2. **p < 0.001, **p < 0.01 and *p < 0.05. Click here for file
