# Supplementary material for: Ring cell migration assay identifies distinct effects of extracellular matrix proteins on cancer cell migration
Source: BMC Res Notes. 2014 Mar 27;7:183. doi: 10.1186/1756-0500-7-183 (PMC3986826; doi:10.1186/1756-0500-7-183)
Supplement: Additional file 1: Figure S1 — Representation of the gap of the U251N glioma cells at 24 h after removal of the cloning ring. [file 1756-0500-7-183-S1.pdf]

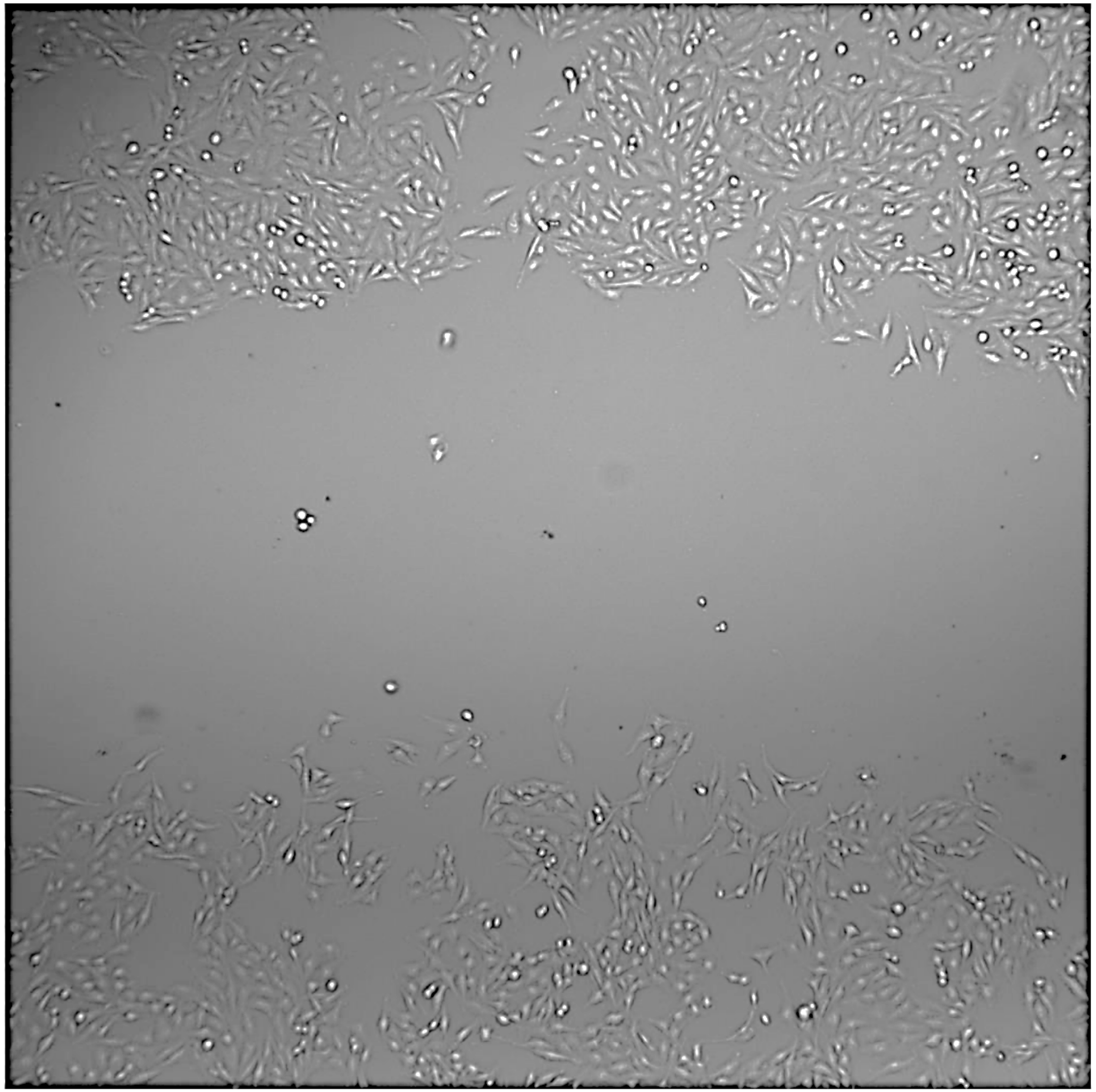

Supplementary Figure 1 – Representation of the gap of the U251N glioma cells at 24h after removal of the cloning ring
