# Supplementary material for: Ring cell migration assay identifies distinct effects of extracellular matrix proteins on cancer cell migration
Source: BMC Res Notes. 2014 Mar 27;7:183. doi: 10.1186/1756-0500-7-183 (PMC3986826; doi:10.1186/1756-0500-7-183)
Supplement: Additional file 2: Figure S2 — Slope as an indicator of distinct effects of extracellular matrices on cell migration. Ring cell migration assay was performed on U87, U251N, MDAMB231, MCF7 and HeLa cells grown on the indicated extracellular matrices and the slope was measured from Figure 5. One-way ANOVA Dunnett’s Multiple Comparison Test: Compare all columns vs. Control, N = 2. **p < 0.001, **p < 0.01 and *p < 0.05. [file 1756-0500-7-183-S2.pdf]

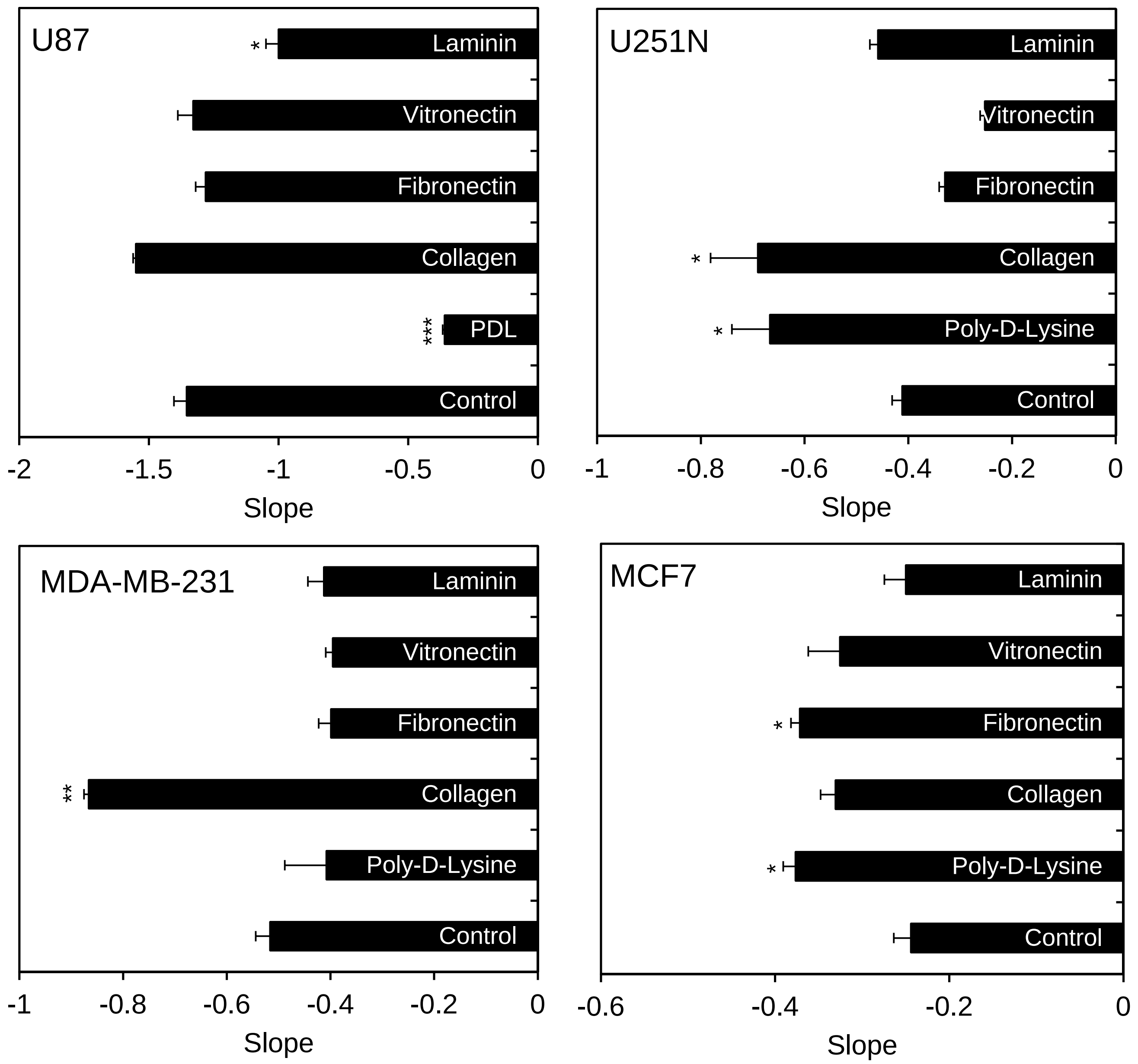

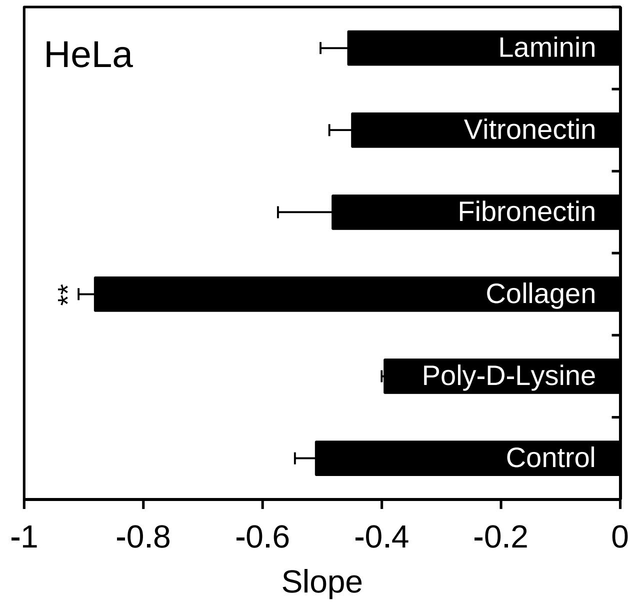

Supplementary Figure 2 - Slope as an indicator of distinct effects of extracellular matrixes on cell migration. U87, U251N, MDA-MB231, MCF7 and HeLa cells grown on indicated extracellular matrixes were perfomed with ring cell migration assay and the slope were measured from Figure 5. One-way ANOVA Dunnett‘s Multiple Comparison Test: Compare all columns vs. Control, N = 2. ** $p<0.001$, ** $p<0.01$ and * $p<0.05$.
